# Not All Sore Throats Are Pharyngitis

**DOI:** 10.5811/cpcem.2017.5.33316

**Published:** 2017-09-29

**Authors:** Adam Sadowski, Joesph Dougherty

**Affiliations:** Ohio Valley Medical Center, Department of Emergency Medicine, Wheeling, West Virginia

## Abstract

Presentations to the emergency department (ED) can often appear to be simple and common. Only when a physician begins to think outside the box when confronting what seems to be a simple condition can a life-threatening situation be avoided. This case provides insight into a common chief complaint seen everyday in the ED – “sore throat.” Not until the patient was seen on several subsequent encounters was a further work-up initiated and the diagnosis made.

## INTRODUCTION

A six-year-old female presented to an emergency department (ED) with chief complaint of sore throat. On initial encounter she was diagnosed with viral pharyngitis and sent home. She presented several days later with continued sore throat and was again diagnosed with viral pharyngitis and discharged home. It wasn’t until a third visit that a more thorough work-up was completed and demonstrated suppurative thyroiditis. This rare medical condition is due to a bacterial infection located in the thyroid gland. Treatment requires antibiotic therapy and sometimes surgery.

## CASE REPORT

A six-year-old female presented to a small rural ED with her mother for a four-day history of sore throat and fever. Fever at home was reported as high as 102 degrees Fahrenheit. The mother had been treating her child’s symptoms with over-the-counter acetaminophen and cold medicine. There was additional report of ear pain and a mild cough. There were no complaints of abdominal pain, urinary symptoms, or rash. Immunizations were reported as up to date. The patient had recently been seen by her pediatrician and had a rapid strep screen that was negative. There was no significant past medical history, surgical history, or allergies. The child took no scheduled medications at home. Reviews of systems were negative.

On initial presentation, triaged vitals were temperature 102.7 degrees Fahrenheit, heart rate 112 beats per minute (bpm) and blood pressure 97/72 mmHg. The child was awake, alert, and interactive. On exam, no significant posterior pharyngeal erythema or tonsillar exudates were noted. Bilateral ear canals were patent with no tympanic membrane abnormalities. There were no other noteworthy physical exam findings. Based on the history and physical exam, the patient’s pharynx was re-swabbed for rapid strep and she was given ibuprofen for her fever. The rapid strep throat screen resulted negative. She was diagnosed with viral pharyngitis and was discharged home with follow-up with her pediatrician in two days.

Six days later the patient presented a second time. This time the chief complaint was worsening sore throat and swollen hard neck. Temperature on arrival was 101.6 degrees Fahrenheit, heart rate 156 bpm, blood pressure 112/83 mmHg, and respiratory rate 20 breaths per minute. Pertinent exam findings were anterior neck that was firm and with fullness with palpation. Diffuse cervical lymphadenopathy was present. Patient was able to flex and extend her neck normally. No tonsillar exudates or erythema noted. Tympanic membranes were clear with patent canals bilaterally. Another rapid strep screen and culture were collected and recorded as negative. Radiographs of the chest and soft tissue of the neck were read by radiology as no acute findings. A mono-spot test resulted negative. Complete blood count (CBC) showed white blood cells (WBC) 19.1 K/uL; high end of assay was 11,000 K/uL. The patient was diagnosed with pharyngitis and was administered dexamethasone and amoxicillin/clavulanate. She was discharged to home with a prescription for amoxicillin/clavulanate and prednisolone and directed to follow up with her pediatrician.

Several hours later the attending emergency physician contacted the family to inquire how the child was doing post discharge. When the mother reported that her child was vomiting, she was told to return to the ED. A third time to the ED, seven hours after recent discharge, the patient presented clammy and diaphoretic. Heart rate was 58bpm, blood pressure 123/61 mmHg, temperature 97.9, and respiratory rate 16 breaths per minute. CBC showed WBC 21.1 K/uL. Basic metabolic panel was within normal limits of assay, as was a urinalysis. Anti-streptolysin-O blood test was reported as negative. Thyroid-stimulating hormone was ordered and reported as undetectable and free T4 2.29 NG/DL elevated for this assay.

**Image 1:**
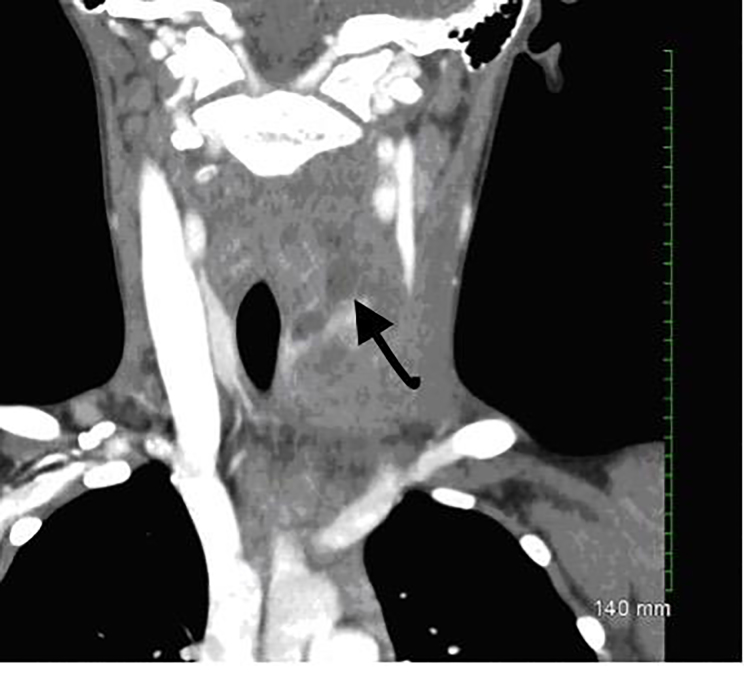
Coronal computed tomography of the neck demonstrating multiloculated heterogeneous fluid (arrow) in the left thyroid lobe

**Image 2:**
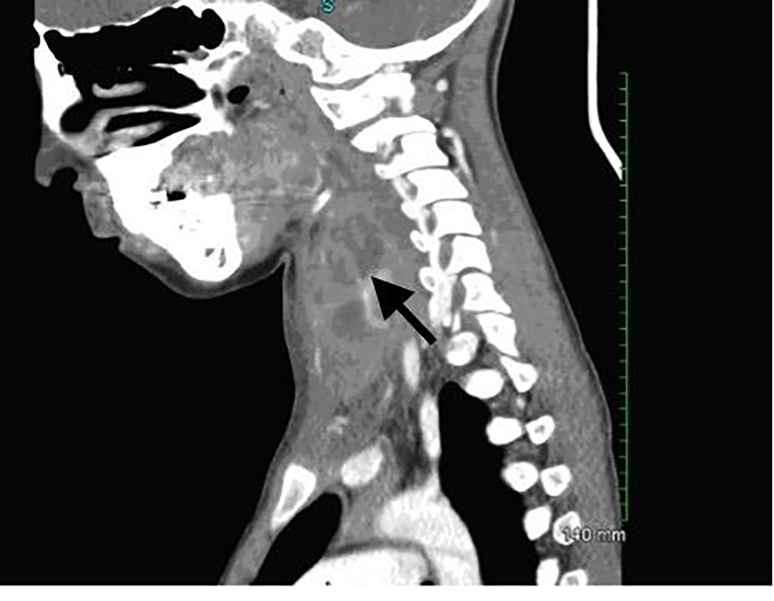
Saggital computed tomography of the neck demonstrating multiloculated heterogeneous fluid (arrow) in the left thyroid lobe.

Computed tomography (CT) of the soft tissue of the neck was completed and read by radiology as multiloculated heterogeneous fluid and presence of a soft tissue lesion in the left thyroid lobe measuring 2.8 × 3.5cm in axial cross-section and about five centimeters in craniocaudal dimension (Images 1 and 2). Findings were most compatible with acute suppurative thyroiditis.

## DISCUSSION

Suppurative thyroiditis is a rare medical condition caused by infection of the thyroid gland. Fewer than 100 cases are reported in the literature each year.^1^ The condition can be life-threatening. The thyroid gland is usually resistant to infections due to a high blood supply, rich lymphatics, iodine content, presence of a tough capsule, and anatomical positioning.^2^ The most common cause of suppurative thyroiditis is from a pyriform sinus fistula connecting the pharynx to the thyroid tissue resulting from a third or fourth brachial arch anomaly during embryonic development, found in up to 70% of cases.^3^ The left thyroid lobe is affected in roughly 90% of cases.^2^

**CPC-EM Capsule**

What do we already know about this clinical entity?

Suppurative thyroiditis is a rare medical condition, most often caused by a pyriform sinus fistula connection from the pharynx to the thyroid gland. The most common presenting symptoms are sudden onset of pain and warmth near the anterior neck. The preferred imaging modality is ultrasound or CT. Treatment includes antibiotic therapy and surgical correction.

What makes this presentation of disease reportable?

This presentation is reportable because of the disease anomaly. The patient presented three times to an ED and once with her primary care physician, before the diagnosis was made. On most of those visits the chief complaint, “sore throat,” was focused on rapid-strep tests that resulted negative. It wasn’t until the child presented with concerning vital signs that a more thorough work-up was initiated. This case reiterates that not all “sore throats” are simple pharyngitis. When the exam and clinical tests do not match, or if the patient is not improving, then a broader differential should be considered.

What is the major learning point?

The emergency physician must keep an open mind and avoid tunnel vision when evaluating patients who present for the same complaint multiple times. Rare and dangerous medical conditions may manifest as simple complaints such as vague abdominal pain, chest pain or, in this case, sore throat. Performing the same test with similar results on multiple visits can miss life-threatening pathology.

How might this improve emergency medicine practice?

This case exposes a rare condition that presents as a common complaint but is really a life- threatening diagnosis. There are only a handful of cases seen each year. Understanding the presentations, mistakes of previous providers, diagnosis, and proper treatment can prevent medical complications and most importantly provide quality care.

Most common bacteria found to cause suppurative thyroiditis in children is *Staphylococcus aureus, Streptococcus pyrogenes, S. epidermidis,* and *Streptococcus pneumonia,* in descending order of frequency.^2^ The disease is not isolated to bacteria. Viruses, such as measles, influenza, enterovirus, Epstein-Barr, adenovirus, cytomegalovirus, echovirus and mumps, can also cause infection.^2^

The most common presenting symptom is sudden onset of pain with firm, tender, red, and warm swelling in the anterior aspect of the neck.^4^ Unfortunately, the risk of recurrent infections from this connection is not well studied. Neck pain is usually unilateral and will radiate to the ears. A detailed focused neck exam is imperative when examining the patient. Differential diagnosis should include adenoma, goiter, or cervical lymphadenitis.^2^ The patient from this case demonstrated an overactive thyroid; however, most cases are euthyroid.

The preferred imaging method for diagnosing this condition is ultrasound.^5^ If visualized, the abscess should be aspirated or surgically drained. Ultrasound will most commonly reveal unilobular swelling with an ill-defined heterogeneous hypoechoic lesion^5^. CT of the neck and magnetic resonance imaging are generally not needed unless ultrasound is not available (as in our case), or if the clinician suspects a mediastinal etiology.^6^ Sometimes radiographs of the neck can be useful in looking for evidence of tissue edema or subcutaneous air.

The management of suppurative thyroiditis is not well studied since there are not many cases diagnosed. Primary therapy is surgical correction of the fistula tract anomaly, if seen on imaging, and drainage of the abscess. Antibiotic therapy should be initiated to include broad-spectrum coverage with clindamycin, amoxicillin-clavulanate, piperacillin with tazobactam, carbapenems, or metronidazole plus either a macrolide or amoxicillin.^2^

## CONCLUSION

The importance of this case is to recognize that not every sore throat is simply pharyngitis. Other causes should be included in the differential. The patient presented febrile with concerning vital signs and was examined three times before the diagnosis was established. Most rapid strep screens today are 95% specific and between 70–90% sensitive for Group A streptococcus.^7^ Even if the patient has a negative strep screen the clinician should look to other etiologies when the physical exam does not match the diagnostic test. In this case not once were signs of strep pharyngitis, such as pharyngeal erythema or exudates, documented. Instead, signs of swollen hard neck and anterior neck fullness were made. If the neck exam demonstrates findings of more concerning etiologies, suppurative thyroiditis should be included the differential. Ultrasound, which is relatively inexpensive and easy to perform and will not expose the child to unnecessary radiation, should be used if available.
